# Validation of Two Global and Generic Quality of Life Questionnaires for Population Screening: SCREENQOL and SEQOL

**DOI:** 10.1100/tsw.2003.29

**Published:** 2003-05-19

**Authors:** Soren Ventegodt, Eskild W. Henneberg, Joav Merrick, Jes S. Lindholt

**Affiliations:** The Quality of Life Research Center, Copenhagen, Denmark

**Keywords:** quality of life, screening, questionnaires, validation, Nottingham Health Profile, Sickness Impact Profile, human development, Denmark

## Abstract

Population screening may harm quality of life (QoL), and traditional health-related QoL tools could be inadequate to evaluate this risk. Two global and generic QoL instruments were developed for studying the QoL consequences of screening (SCREENQOL), and QoL variation in a normal population (SEQOL). SCREENQOL and SEQOL (Self-Evaluation of Quality of Life Questionnaire) are self-administered questionnaires with items rated on 5-point Likert scales. SCREENQOL consists of 21 items measuring QoL across 6 different dimensions based upon validated QoL questionnaires. SEQOL consists of 317 items measuring QoL across 8 different dimensions, based on an integrative theory of QoL, a theoretical framework from a Danish QoL survey involving 7,222 persons 31 to 33 years old. For further validation, SEQOL and SCREENQOL were sent to 2,460 persons 18 to 88 years old randomly selected from the Danish Central Person Register together with Nottingham Health Profile (NHP) and Sickness Impact Profile (SIP). For SCREENQOL and SEQOL, test-retest reliability correlation was both >0.8, Cronbach's alpha was 0.65 and 0.75, correlation (r) to NHP was 0.67 and 0.49, to SIP 0.46 and 0.27, respectively (p

### INTRODUCTION

Population screening is known to be able to cause harmful psychological consequences. Consequently, evaluation of the consequences must be made before permanent screening of a new field can be recommended[1,2,3,4,5]. The measurement of the subjective dimensions of health and quality of life (QoL) in medical research has long been an area of considerable interest. More than 10,000 articles have been written on QoL, health-related QoL, or self-evaluated health, and thousands of new studies are published each year. There now exists such an array of health measures, all with their own individual strengths and weaknesses, that it is increasingly difficult to determine which is most valid for a particular study. Furthermore, many of the instruments are specific to a particular disease group. However, no relevant questionnaire specific for consequences of mass screening seems available. This may be due to the difficult methodological demands of such a questionnaire because it ought to be global and generic, but still focus on known and potential side effects of the offer of screening.

The term "global" means that it measures the overall QoL, and "generic" means that it can be used on everybody, despite culture, age, sex, or heath status. This paper reports on the development and validation of two global and generic QoL measures. First is a questionnaire developed to study the potential effect on QoL caused by population screening (SCREENQOL). In order to achieve a high-quality global and generic QoL questionnaire for the validation of the SCREENQOL, a large questionnaire developed at the University of Copenhagen for the Danish Quality of Life Research Study was also validated in this study (SEQOL).

### METHODS

#### Quality of Life Questionnaire for Population Screening (SCREENQOL)

*A QoL questionnaire was developed based on existing, validated questionnaires focusing on the earlier reported psychological side effects of screening[6,7,8,9,10].* By adding a number of psychosocial items, a more generic concept of QoL to the questionnaire was achieved. In order to be able to achieve representative data from nonattenders to screening, and data from other relevant groups that easily can be generalised, high response rates are preferable in studies concerning the psychological consequences of screening. Consequently, a relatively small number of items were requested. Therefore, the questionnaire ended up only comprising 21 items in 6 categories: general self-assessed QoL, emotional state, physical health, psychosomatic distress, social and family function, and marriage. The questionnaire was initially tested without reminders on 1,490 males 65 to 73 years old; 71% responded. Of these, a retest was conducted on 106 persons more than half a year after the initial administration of the questionnaire. 85% of the respondents fulfilled the questionnaire completely[11]. For further validation, a study was designed to compare SCREENQOL with golden standards of health-related QoL (Nottingham Health Profile [NHP] and Sickness Impact Profile [SIP]) as global and generic QoL. There are several theories concerned with global/overall QoL, however; only one questionnaire integrates these philosophies into one.

#### Self-Evaluation of Quality of Life Questionnaire (SEQOL)

*A study group consisting of doctors, nurses, social workers, philosophers, and others interested in health and QoL examined various QoL theories and designed a methodological framework for the measurement of QoL.* A methodology was proposed that defined construct criteria, which the questionnaire and its items should satisfy to secure construction validity; a definition of QoL, a coherent philosophy of human life, a theory that deduces specific questions from the philosophy of life, quantifiable response options, and meaningfulness among others[12].

Eight of such theories were identified from the philosophical, social science, psychological, and medical traditions. They were merged into an integrative theory of QoL organising the eight theories into a spectrum ranging from subjective (self-evaluated) to objective (externally evaluated) QoL and spanning a core of theories that consider QoL as deriving from human nature or human existence itself (existential theories). The questionnaire was revised through pilot studies and qualitative interviews 20 times before it was in broad agreement with the conceptual definition. The final questionnaire was initially tested in a pilot study on 7,220 persons[13,14]; 80% said they could express their QoL “well” or “very well”. A retest after 28 days took place in 130 randomly selected respondents .

### Combined Validation Study

A representative sample of 18- to 88-year-old people from the Danish population, randomly selected from the Central Persons Register (CPR), received a 500-item questionnaire incorporating SCREENQOL, NHP, SIP, and SEQOL; 1,100 responded without use of reminders.

The sensitivity was calculated as the number of respondents needed to detect a 3, 10, and 20% difference in the QoL, respectively. Internal consistency was evaluated by the Cronbach´s alpha method, and criteria validation was conducted through correlations with NHP and SIP using Pearson´s test.

## RESULTS

### External Reliability and Sensitivity

The reliability of the SCREENQOL and SEQOL was examined through test-retests. The results were examined across all dimensions using correlation analysis (Table 1). All dimensions and the total QoL scores maintained high correlation above 0.80 with the initial ratings.

The sensitivity of the instrument, defined as its ability to detect real differences in QoL, is shown in Table 2. Respectively, 61 and 111 respondents were needed to detect a 3% difference in QoL using SCREENQOL or SEQOL.

### Internal Reliability

By calculating Cronbach’s alpha coefficients (standardised variables) for each of the dimensions, the internal reliability was tested (Table 4). The overall coefficients were 0.68, 0.73, 0.78, and 0.85 for SCREENQOL, SEQOL, NHP, and SIP, respectively.

Correlation (r) to NHP was 0.67 and 0.49 for SCREENQOL and SEQOL, respectively, and 0.46 and 0.27 to SIP, respectively ($p < 0.05$). When only the results from the respondents reporting ill-health defined as receiving treatment by a doctor for one or more medical complaints were used, the correlations became stronger, r = 0.41 and 0.68, respectively.

## DISCUSSION

We had some difficulties determining how to validate the new questionnaires. As they were quite different from the often-used questionnaires like SIP and NHP, we wondered if it was still

**TABLE 1**
**Reliability, SEQOL (Test-Retest D28) and SCREENQOL (Test-Retest D180)**

| | Number of Respondents (n) | Correlation (r) |
|---|---|---|
| **Dimension (SEQOL)** | | |
| Well being | 116 | 0.60 |
| Satisfaction with life | 116 | 0.65 |
| Happiness | 116 | 0.72 |
| Family, work, and leisure | 106 | 0.65 |
| Fulfillment of needs | 102 | 0.73 |
| Satisfaction with relationships | 106 | 0.65 |
| Realising life potential | 102 | 0.72 |
| Objective factors | 104 | 0.86 |
| Overall QoL | 117 | 0.89 |
| **Dimension (SCREENQOL)** | | |
| Self-assessed quality of life | 106 | 0.73 |
| Emotional | 106 | 0.73 |
| Physical health | 106 | 0.78 |
| Psychosomatic distress | 106 | 0.80 |
| Social/family | 106 | 0.78 |
| Marriage | 106 | 0.89 |
| Overall QoL | 106 | 0.86 |

appropriate to use the SIP and NHP to establish their validity. It seemed that SCREENQOL and SEQOL could validate each other, but to convince the medical society that SCREENQOL and SEQOL actually measure QoL in any relevant dimension we agreed that we had to include SIP and NHP in the validation also.

Using the two measures in three QoL studies including almost 10,000 people showed us that they are indeed suitable for population screening[11,12,13,14]. Most widely used is the SEQOL and most of the respondents reported that they felt that the SEQOL questionnaire expressed their QoL.

### External Reliability

Both SCREENQOL and SEQOL showed high correlations of reproducibility and sensitivity, both important factors for monitoring individual changes. The interval between the tests has to be long enough for the respondents to forget the answers, and short enough to be retested without changes in QoL. SCREENQOL was retested after a longer interval than usual, but still a strong correlation was noticed. If the QoL changed during the period, the strong correlation could be explained by poor sensitivity. However, only 61 to 111 respondents would be needed to detect 3% differences in QoL concerning SCREENQOL and SEQOL, respectively. Such a small difference is seldom clinically important, and even fewer would be needed with the use of paired observations.

**TABLE 2**
**Sensitivity for SEQOL and SCREENQOL**

| | Number of Respondents Needed to Detect Given Difference | | | Standard Deviation |
|---|---|---|---|---|
| | 3% | 10% | 20% | |
| **Dimension (SCREENQOL)** | | | | |
| Self-assessed quality of life | 249 | 24 | 7 | 17.0 |
| Emotional | 121 | 13 | 5 | 11.8 |
| Physical health | 160 | 16 | 5 | 13.6 |
| Psychosomatic distress | 105 | 11 | 4 | 11.0 |
| Social and family situation | 211 | 21 | 7 | 15.7 |
| Marriage | 594 | 55 | 15 | 26.3 |
| Overall SCREENQOL | 61 | 7 | 3 | 8.4 |
| **Dimension (SEQOL)** | | | | |
| Well being | 236 | 23 | 7 | 16.6 |
| Satisfaction with life | 234 | 23 | 7 | 16.5 |
| Happiness | 204 | 20 | 6 | 15.4 |
| Fulfillment of needs | 134 | 14 | 5 | 12.4 |
| Family, work, and leisure | 120 | 12 | 5 | 11.8 |
| Satisfaction with relationships | 89 | 10 | 4 | 10.1 |
| Realising life potential | 113 | 12 | 4 | 11.4 |
| Objective factors | 230 | 22 | 7 | 16.4 |
| Overall SEQOL | 111 | 12 | 4 | 11.3 |

Sensitivity: standard deviation(s) presumed unknown. Hypotheses are $H_0$ = m1=m2 and $p < 0.05$.

### Internal Reliability

A value of Chronbach´s alpha below 0.45 normally signifies a lack of focus, whereas a high value of alpha above 0.85 normally signifies either redundancy or a very high internal consistency[15]. Consequently, the coefficients of SCREENQOL and SEQOL are in the range that is normally preferred. However, the internal consistency seemed a bit lower for SCREENQOL, but this was expected because the number of items are used in calculating Chronbach´s alpha favouring more items. SCREENQOL was designed to have few items.

Besides each other, we compared the instruments with NHP[16,17] and the SIP[18] — two widely used health-related QoL instruments that have both demonstrated acceptable levels of validity over a range of patient groups[9,17], and both have been translated with valid back translation.

To see whether the underlying fundamental dimensions of health, illness, social function, etc. examined in SCREENQOL and SEQOL were the same as those dimensions traditionally examined in the medical world, we conducted a study of criteria validation (Table 4). The low correlation between the instruments and SIP is probably due to the fact that SIP was designed to assess the impact of illness on everyday life, and though it has been shown to be applicable across diverse demographic groups, it seemingly loses validity in healthy groups, whereas the NHP,

**TABLE 3**
**Internal Reliability for SEQOL and SCREENQOL**

| | | Cronbach's alpha | |
|---|---|---|---|
| | n | Raw variables | Standardized v. |
| **Dimension (SEQOL)** | | | |
| Well being | 1066 | 0.881 | 0.894 |
| Satisfaction with life | 1066 | 0.877 | 0.891 |
| Happiness | 1063 | 0.878 | 0.893 |
| Family, work, and leisure | 1001 | 0.879 | 0.890 |
| Fulfillment of needs | 1002 | 0.877 | 0.890 |
| Satisfaction with relationships | 838 | 0.878 | 0.886 |
| Realising life potential | 1067 | 0.883 | 0.894 |
| Objective factors | 950 | 0.912 | 0.918 |
| Overall SEQOL | | 0.706 | 0.748 |
| **Dimension (SCREENQOL)** | | | |
| Self-assessed quality of life | 1092 | 0.381 | 0.546 |
| Emotional | 1094 | 0.406 | 0.544 |
| Physical health | 1095 | 0.402 | 0.548 |
| Psychosomatic distress | 1095 | 0.439 | 0.564 |
| Social/family | 1093 | 0.551 | 0.681 |
| Marriage | 1084 | 0.708 | 0.726 |
| Overall SCREENQOL | | 0.528 | 0.653 |
| **Dimension (NHP)** | | | |
| Energy level | 1012 | 0.709 | 0.728 |
| Pain | 989 | 0.731 | 0.749 |
| Emotional reactions | 976 | 0.700 | 0.715 |
| Sleep | 1007 | 0.725 | 0.757 |
| Social isolation | 1005 | 0.728 | 0.759 |
| Physical abilities | 1009 | 0.724 | 0.749 |
| Overall NHP | | 0.755 | 0.776 |
| **Dimension (SIP)** | | | |
| Sleep and rest | 994 | 0.791 | 0.848 |
| Eating | 962 | 0.797 | 0.854 |
| Work | 442 | 0.792 | 0.840 |
| Home management | 962 | 0.767 | 0.826 |
| Recreation and pastimes | 904 | 0.792 | 0.840 |
| Ambulation | 960 | 0.770 | 0.824 |
| Mobility | 970 | 0.773 | 0.826 |
| Body care and movement | 947 | 0.789 | 0.827 |
| Social interaction | 832 | 0.762 | 0.823 |
| Alertness behaviour | 974 | 0.771 | 0.832 |
| Emotional behaviour | 963 | 0.782 | 0.839 |
| Communication | 990 | 0.793 | 0.846 |
| Overall SIP | | 0.797 | 0.847 |

**TABLE 4**
**Criteria Validation, Whole Sample and Patients Currently Receiving Medical Treatment**

| | NHP | | | SIP | | | SCREENQOL | | | SEQOL | | |
|---|---|---|---|---|---|---|---|---|---|---|---|---|
| | **r** | **p** | **n** | **r** | **p** | **n** | **r** | **p** | **n** | **r** | **p** | **n** |
| **Whole sample** | | | | | | | | | | | | |
| NHP | * | * | * | 0.650 | 0.0001 | 499 | 0.672 | 0.0001 | 900 | –0.491 | 0.0001 | 898 |
| SIP | 0.650 | 0.0001 | 499 | * | * | * | 0.457 | 0.0001 | 677 | –0.265 | 1E-04 | 680 |
| SCREENQOL | 0.672 | 0.0001 | 900 | 0.457 | 1E-04 | 677 | * | * | * | –0.604 | 1E-04 | ### |
| SEQOL | –0.491 | 0.0001 | 898 | –0.26 | 1E-04 | 680 | –0.60 | 0.0001 | 1201 | * | * | * |
| **Patients currently receiving medical treatment** | | | | | | | | | | | | |
| NHP | * | * | * | | 0.0001 | | | 0.0001 | | | 0.0001 | |
| SIP | 0.698 | 0.0001 | 110 | * | * | * | | 0.0001 | | | 0.0001 | |
| SCREENQOL | 0.740 | 0.0001 | 217 | | 0.0001 | | * | * | * | | 0.0001 | |
| SEQOL | –0.551 | 0.0001 | 216 | | 0.0001 | | | 0.0001 | | * | * | * |

Table gives the Pearson correlation coefficient (r), the test value (p), and number of respondents (n) for each QoL instrument.

which shows a moderate correlation, has affirmed that it can differentiate severe from nonsevere illness groups or well groups[16,18].

Whereas the NHP and SIP were designed to be sensitive to changes in health status in very sick patients, the SCREENQOL and SEQOL were designed to provide a suitable global, generic QoL measure for a large prospective quality of life studies — SCREENQOL especially for population screening involving healthy persons. However, SCREENQOL (r = 0.65) and SEQOL both have a strong linear connection with self-evaluated health[13,14]. The linear relationship suggests that the instruments can be used with population groups regardless of health status, allowing one to compare healthy and ill populations alike. Additionally, in the case of prospective studies, it allows one to monitor people as they move through various health states.

Why is there a need for QoL questionnaires like SCREENQOL and SEQOL, when the world is already so packed with questionnaires? The reason is that we need good QoL questionnaires to provide us with exact and detailed information on the QoL of the population at large. Either the existing questionnaires are very brief, or they are not as global and generic as one would like, but often usable only in the context of one specific disease.

Why is it necessary to have these two measures? SCEENQOL is based on intuition and experience from the long tradition of measuring QoL in medicine. SEQOL is based on an integrative theory for QoL. These two different, but equally important, approaches seem to give similar numbers, which is important as it indicates that QoL actually exist in the real world and is a measurable phenomena. Still many health politicians and also some medical professionals seem to doubt that.

In what ways are SCREENQOL and SEQOL different from the existing measures? They are precise and sensitive, global and generic QoL measures designed for measuring the QoL of the general population in all relevant dimensions, usable on almost any group of people or patients, regardless of health status, culture, gender, or age.

### CONCLUSION

SCREENQOL and SEQOL seem to have an acceptable construct validity, external reliability, sensitivity, and internal reliability. However, SCREENQOL tended to have lower internal reliability evaluated by Chronbach´s alpha. This was expected as a consequence of the deliberate lower number of items. They both correlate fairly well to NHP and to some degree with SIP, especially among the deceased population

This was also expected, since NHP and SIP measured health-related QoL, while SCREENQOL and SEQOL measures global/overall QoL.

In all, they both appear valid for researching global QoL in healthy or adult populations with specific diseases regardless of gender, age, or race.

### ACKNOWLEDGEMENTS

We appreciate our sources of support: The 1991 Pharmacy Foundation of 1991, The Goodwill Foundation, The Danish Heart Foundation, J.L. Fonden, Direktør E. Danielsen and Hustrus Fond, Emmerik Mayers Fellowship, Frimodt-Heineken Fond, The Family of Hede Nielsen's Fond, Petrus Andersens Fond, Grosserer C.F. Frederiksens Scholarship for medical students and young doctors, Else & Mogens Wedell-Wedellsborgs Fond, IMK Almene Fond.

### BIOSKETCH

**Søren Ventegodt, M.D.,** is the Director of the the Quality of Life Research Center in Copenhagen, Denmark. He is also responsible for a Research Clinic for Holistic Medicine in Copenhagen and is a popular speaker throughout Scandinavia. He has published numerous scientific, popular articles and a number of books on holistic medicine, quality of life, and quality of working life. His most important scientific contributions are the comprehensive SEQOL questionnaire, the very short QoL5 questionnaire, the integrated QoL theory, the holistic process theory, the life mission theory, and the Danish Quality of Life Research Study at the University Hospital of Copenhagen with the late pediatric Professor Bengt Zachau-Christiansen.

**Eskild W. Henneberg, M.D.,** is former Medical Director of Viborg Sygehus, Denmark and former Chairman of the Board of the Danish Society of Vascular Surgeons. At present, he is the leading Consultant Surgeon in Vascular and Thoracic Surgery at Viborg Sygehus and Chairman of the Board of the Danish Society of Surgeons. He has published over 60 scientific articles, mostly together with Jes S. Lindholt through their decade-long collaboration. His main research topics are abdominal aortic aneurysms (AAA) and lower limb atherosclerosis, especially concerning prevention. His most important scientific contributions are screening for AAA, including consequences in quality of life by screening, and medical treatment of AAA.

**Joav Merrick, M.D., D.MSc.,** is Professor of Child Health and Human Development affiliated with the Zusman Child Development Center and Division of Community Health at the Ben Gurion University, Beer-Sheva, Israel and presently the Medical Director of the Division for Mental Retardation, Ministry of Social Affairs, Jerusalem and the Director of the National Institute of Child Health and Human Development.

He has numerous publications in the field of child and human development, rehabilitation, intellectual disability, disability, health, welfare, abuse, advocacy, and prevention. Dr. Merrick received the Peter Sabroe Child Award for outstanding work on behalf of Danish Children in 1985 and the International LEGO-Prize ("The Children's Nobel Prize") for an extraordinary contribution towards improvement in child welfare and well being in 1987.

**Jes S. Lindholt, M.D., Ph.D.,** is a Board Member of the Danish Society of Vascular Surgery, and a Vascular Trainee at the Department of Vascular Surgery at Rigshospitalet in Copenhagen. He has published over 50 scientific articles and presented the results at prestigious scientific meetings worldwide. Research topics are mainly abdominal aortic aneurysms (AAA) and lower limb atherosclerosis, especially concerning prevention. His most important scientific contributions are screening for AAA, including consequences in quality of life by screening, and medical treatment of AAA. In 1998, he achieved the medical Ph.D. degree at the University of Aarhus with the thesis: "Considerations and Experiences of Screening for Abdominal Aortic Aneurysms", published at FADL´s Forlag, Copenhagen.
